# Proteomic characterisation of triple negative breast cancer cells following CDK4/6 inhibition

**DOI:** 10.1038/s41597-022-01512-1

**Published:** 2022-07-11

**Authors:** Melina Beykou, Mar Arias-Garcia, Theodoros I. Roumeliotis, Jyoti S. Choudhary, Nicolas Moser, Pantelis Georgiou, Chris Bakal

**Affiliations:** 1 Imperial College London, Department of Electrical and Electronic Engineering, Circuits and Systems Group, South Kensington Campus, London, SW7 2AZ UK; 2 Institute of Cancer Research, Division of Cancer Biology, Dynamical Cell Systems, London, SW3 6JB UK; 3 Cancer Research UK Convergence Science Centre, South Kensington Campus, London, SW7 2AZ UK; 4 Institute of Cancer Research, Division of Cancer Biology, Functional Proteomics, London, SW3 6JB UK

**Keywords:** Breast cancer, Targeted therapies, Proteomic analysis

## Abstract

When used in combination with hormone treatment, Palbociclib prolongs progression-free survival of patients with hormone receptor positive breast cancer. Mechanistically, Palbociclib inhibits CDK4/6 activity but the basis for differing sensitivity of cancer to Palbociclib is poorly understood. A common observation in a subset of Triple Negative Breast Cancers (TNBCs) is that prolonged CDK4/6 inhibition can engage a senescence-like state where cells exit the cell cycle, whilst, remaining metabolically active. To better understand the senescence-like cell state which arises after Palbociclib treatment we used mass spectrometry to quantify the proteome, phosphoproteome, and secretome of Palbociclib-treated MDA-MB-231 TNBC cells. We observed altered levels of cell cycle regulators, immune response, and key senescence markers upon Palbociclib treatment. These datasets provide a starting point for the derivation of biomarkers which could inform the future use CDK4/6 inhibitors in TNBC subtypes and guide the development of potential combination therapies.

## Abstract (summary)

**Table:** 

Measurement(s)	Proteomic Profiling
Technology Type(s)	Liquid Chromatography Mass Spectrometry
Factor Type(s)	Palbociclib dosage
Sample Characteristic - Organism	Homo sapiens
Sample Characteristic - Environment	cell culture

## Background & Summary

A distinct characteristic of all cancer types is aberrant proliferation which can be attributed to loss of cell cycle control^1,2^. There are four phases to the cell cycle (G1, S, G2 and M phase) where progression through the cycle is controlled by CDKs in conjunction with cyclins^3,4^. In non-malignant cells, G1 progression is controlled by the CDK4/6 – Cyclin D1 complex which phosphorylates Retinoblastoma protein (Rb)^3–6^. The subsequent release of E2F allows the transcription of E2F target genes which are responsible for G1 progression, such as CDK2 and Cyclin E^3–6^. Therapeutic interventions targeting the cell cycle have been promising candidates, especially for Hormone-receptor positive (HR+) breast cancer subtypes where specific cyclin-dependent kinase (CDK) 4/6 inhibitors, such as Palbociclib and Abemaciclib, have shown a high success rate in patient response^5,7–9^. Whilst both these CDK4/6 inhibitors have selectivity, Abemaciclib has exhibited off-target effects on other CDKs, including CDK9, which may result in subsequent toxicity^10,11^. HR+ breast cancers in particular are considered the ideal target for CDK4/6 inhibitors due to a positive feedback loop between the oestrogen receptor activation and Cyclin D1 overexpression which ultimately leads to a strong dependence on the CDK4/6-Cyclin D1 mechanism for cell cycle progression^3,5,12^.

Palbociclib’s ability to block the cell cycle is largely thought to be Rb-dependent, whereby inhibition of Rb phosphorylation (pRb) results in G1 phase arrest^2,6,13,14^. Resistance to CDK4/6 inhibition can be due to null mutations in RB1, which alleviate dependence on CDK4/6, and may be used as a predictive biomarker for response^13–16^. RB1 expression, however, may not be the only factor in determining Palbociclib sensitivity. In fact, recent studies have shown that the response of breast cancer subtypes to Palbociclib is heavily dependent on the activity of other cell cycle regulators including, CDK2, CDK6, Cyclin D and p16^2,13,14,17–19^. In addition, other signaling pathways may influence sensitivity^16,20–22^. Thus, understanding the basis for Palbociclib sensitivity, and why some cell lines show variable resistance despite expressing functional RB1, is warranted.

A common characteristic of Palbociclib treatment is the induction of a morphologically and phenotypically distinct cell state, which resembles senescence^13,14,17,18,23^. The senescent-like state can be induced in subtypes with functional Rb such as the Triple Negative Breast Cancer (TNBC) cell line, MDA-MB-231^13,15,17,23–26^. This cell state remains poorly understood as studies show inconclusive evidence creating uncertainty as to whether this is stable senescence^5,13–15,23,25^. A set of markers, such as pRb, *β*-galactosidase (GLB1) and, loss of Ki67 and LaminB1, have been identified as hallmarks of senescence although they remain non-exclusive to the senescence phenotype^27,28^.

The field of senescence research has recently uncovered both beneficial and detrimental consequences of the cell phenotype for tumour growth. In particular, the pro-tumorigenic and anti-tumorigenic effects of the Senescence-Associated Secretory Phenotype (SASP) remain a point of debate^24,28,29^. More specifically, despite the inhibition in tumour growth that accompanies senescence induction, additional factors can result in tumour recurrence, apoptosis resistance, tumour dormancy and ultimately metastasis^30–32^. Inflammation via inflammatory cytokines secreted as part of the SASP also play an important role for tumour growth and survival^32,33^. On the other hand, senescence can promote an adaptive immune response and some SASP components may sensitise the cell state to combination therapies^24,32,34,35^. One such example is the combination therapy of an anti-PDL1 agent with Palbociclib, which is currently in clinical trials (PAveMenT, https://clinicaltrials.gov/ct2/show/NCT04360941/). Given the potential importance of the Palbociclib-induced, senescent-like phenotype to the treatment of cancers,we sought to better characterize the cell state.

**Fig. 1:**
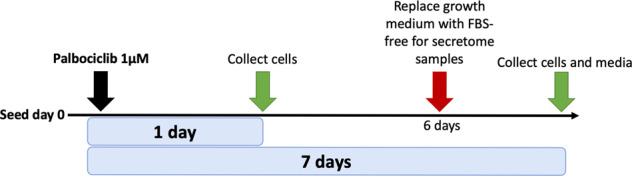
Overview of drug treatment regime with CDK4/6 inhibitor Palbociclib. Samples intended for secretome analysis had their growth medium replaced with fetal bovine serum (FBS) - free growth medium 24 hours prior to collection of samples.

**Fig. 2:**
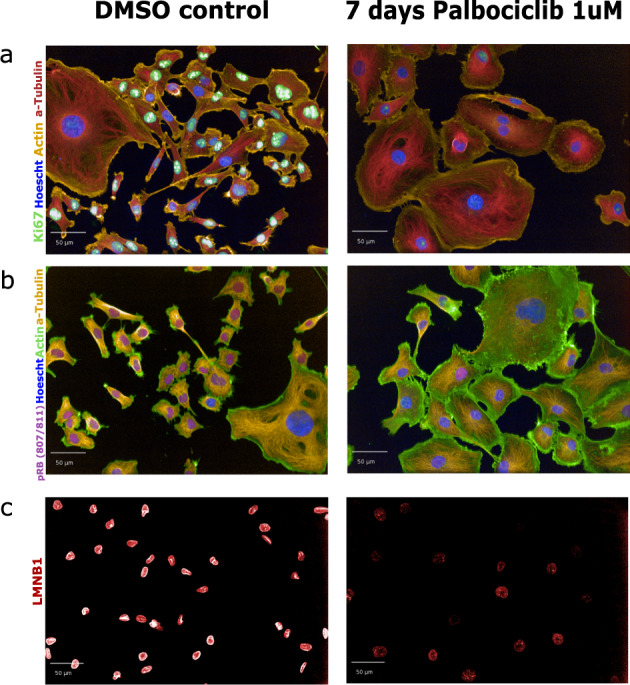
Validation of senescence-like morphology and immunofluorescence staining of key senescence biomarkers. Treatment of MDA-MB-231 cells with 1uM PD0332991 (known as Palbociclib) for 7 days or with an equivalent dose of DMSO as a control. **(a)** Immunofluorescence staining for Ki67. **(b)** Immunofluorescence staining for pRb (807/811). **(c)** Immunofluorescence staining for LMNB1. In all panels actin, a-Tubulin and Hoescht (nucleus) staining is used to image morphological features. All images were taken using the Opera high-throughput microscope (PerkinElmer).

**Fig. 3:**
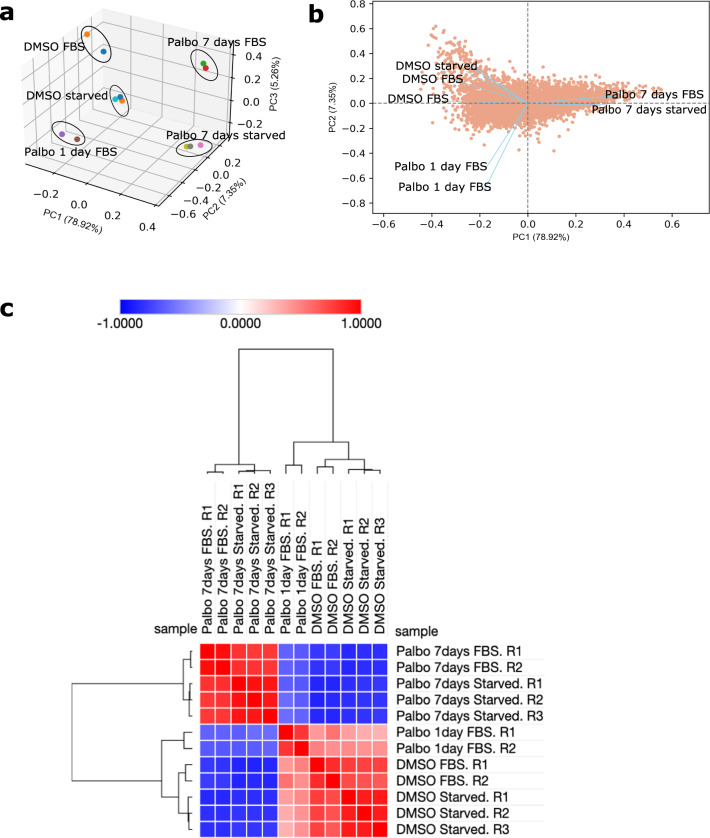
Reproducibility of proteomic samples. **(a)** Principal component analysis (PCA) was performed to illustrate the reproducibility of samples. The PCA plot shows clustering of samples with the same cell culture conditions.The first 3 principal components (PCs) contribute to 91.53% of the variation in the data. **(b)** A PCA biplot illustrating the PC loadings and the first two principal components, PC1 and PC2. The angle between vectors shows that Palbo 7 days FBS and Palbo 7 days starved samples are closely correlated. DMSO starved and DMSO FBS samples are correlated to eachother and have a low correlation with all Palbocicib-treated samples. The PCA analysis was carried out using the bioinfokit package in Python version 3.8.8^55^. **(c)** A similarity matrix (Pearson correlation) of total protein scaled abundances which has been subjected to hierarchical clustering (Euclidean distance with complete linkage), to reveal that samples of the same conditions cluster together on the matrix. Thus, the samples of the same condition in question are highly correlated. The Phantasus server was used for this analysis (https://artyomovlab.wustl.edu/phantasus/).

**Fig. 4:**
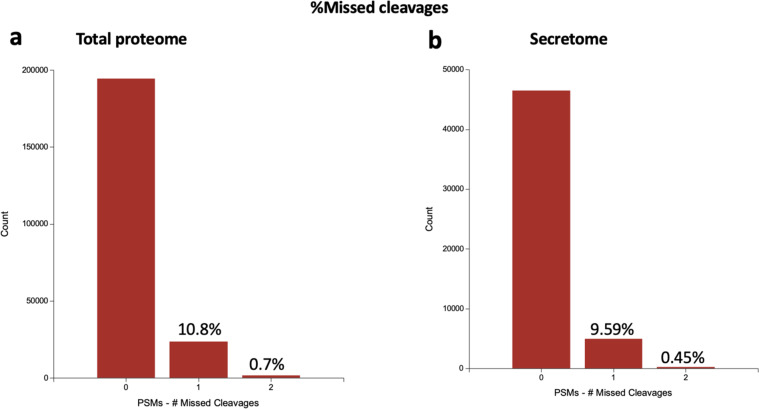
Technical validation of mass spectrometry analysis. The percentage of missed cleavages through trypsin digestion showing reproducibility between the total proteome **(a)** and secretome **(b)** analysis. PSMs = peptide spectrum matches.

**Fig. 5:**
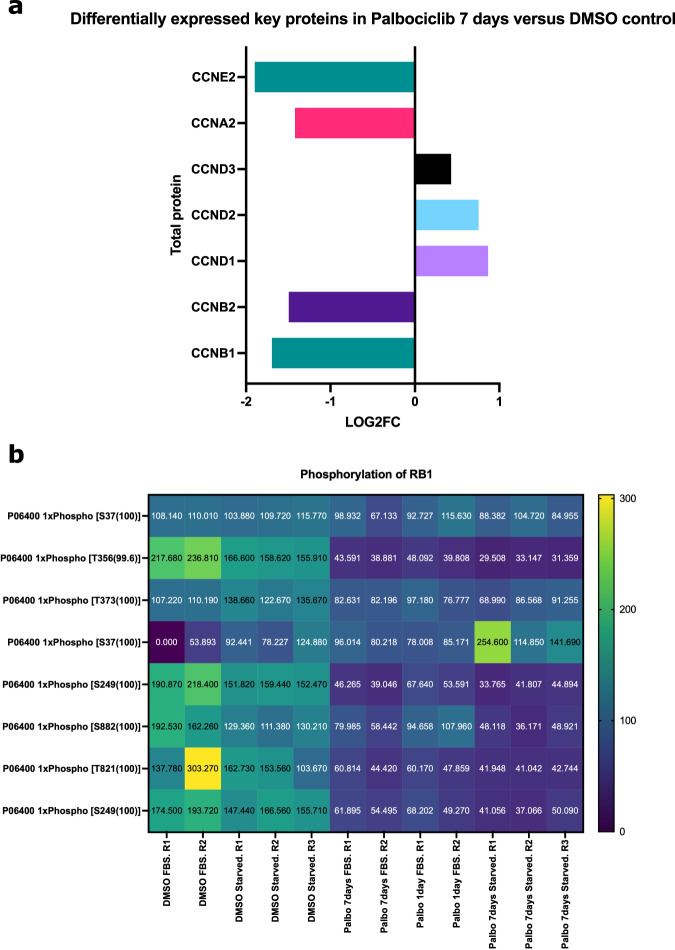
Validation of key senescence markers in total proteome and phosphoproteome. (**a**) Log2FC of cyclins significantly differentially expressed in Palbociclib-treated MDA-MB-231 cells, both FBS and starved samples, versus their respective DMSO controls. Log2FC was calculated using a one sample t-test and subsequent results were filtered for q-value < 0.05 using Perseus software^39^. (**b**) Heatmap of phosphorylation sites of RB1 (pRB) showing a decrease in RB1 phosphorylation at multiple sites after Palbociclib treatment.Log2FC = Log2 Fold Change.

**Fig. 6:**
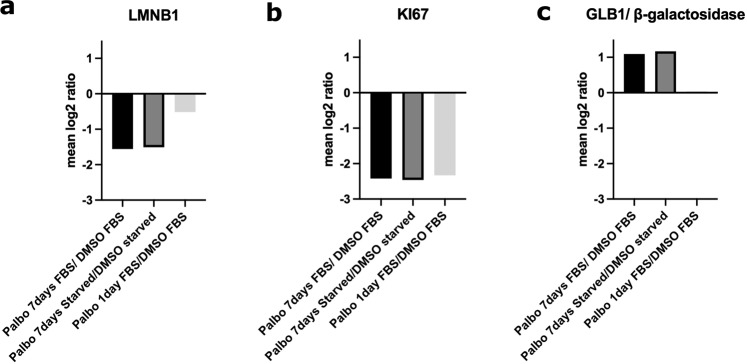
Validation of key senescence markers in total proteome and phosphoproteome. **(a)** Mean Log2 ratio of LMNB1 protein abundance of Palbociclib-treated cells versus their respective controls. **(b)** Mean Log2 ratio of KI67 protein abundance of Palbociclib-treated cells versus their respective controls. **(c)** Mean Log2 ratio of GLB1 protein abundance of Palbociclib-treated cells versus their respective controls.

**Fig. 7:**
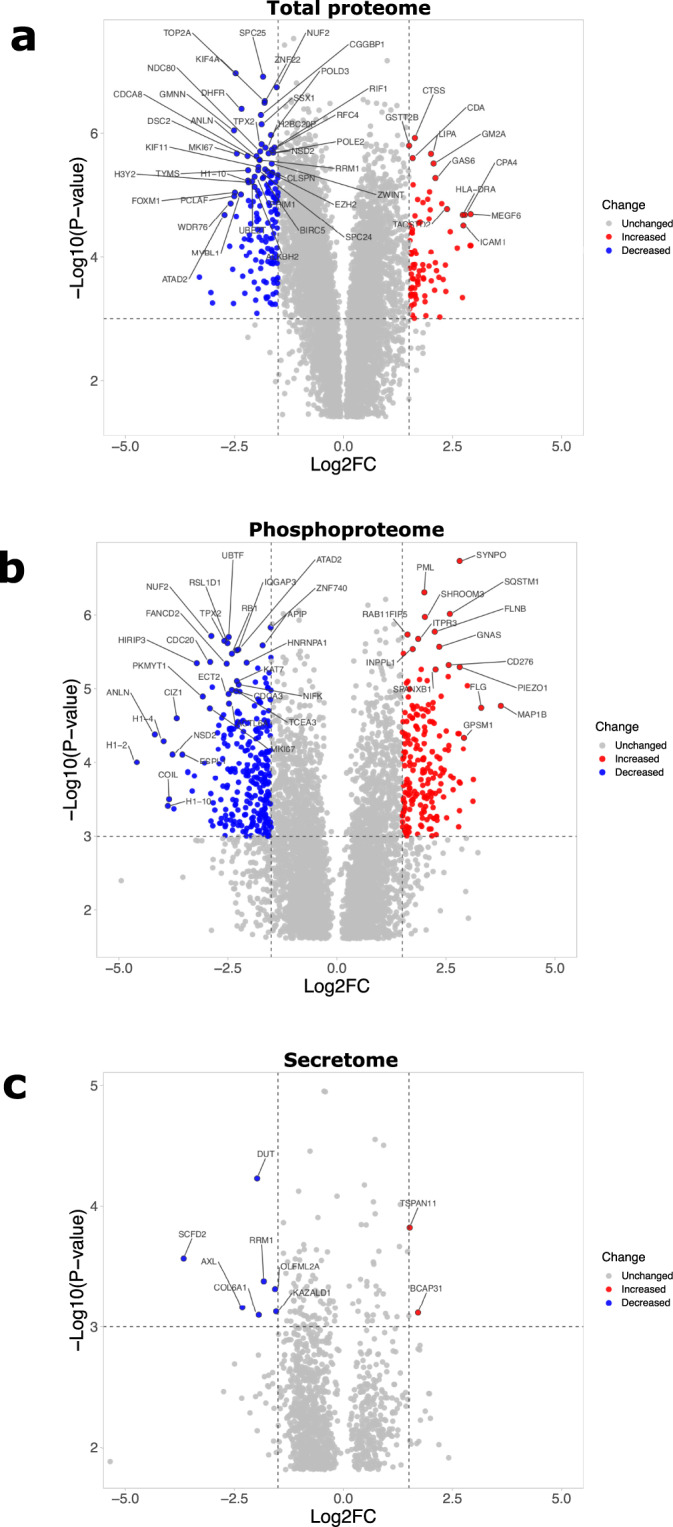
Significantly upregulated and downregulated proteins upon 7 days Palbociclib treatment of MDA-MB-231. **(a)** Proteins with significantly different expression in total proteome of MDA-MB-231 7 days Palbociclib-treated cells (both FBS and starved samples) as compared to their respective DMSO-treated controls. **(b)** Significantly different phosphorylation profile of proteins in MDA-MB-231 7 days Palbociclib-treated cells (both FBS and starved samples) as compared to their respective DMSO-treated controls. **(c)** Significantly different expression of proteins in secretome of 7 days Palbociclib-treated versus DMSO-treated MDA-MB-231 cells. In all cases, one sample t-test was performed and subsequent results were filtered for q-value < 0.05 using Perseus software^39^. Top 50 most significant hits were calculated with the Manhattan metric using VolcaNoseR (R2 mirror)^40^. Log2FC = Log2 Fold Change.

**Fig. 8:**
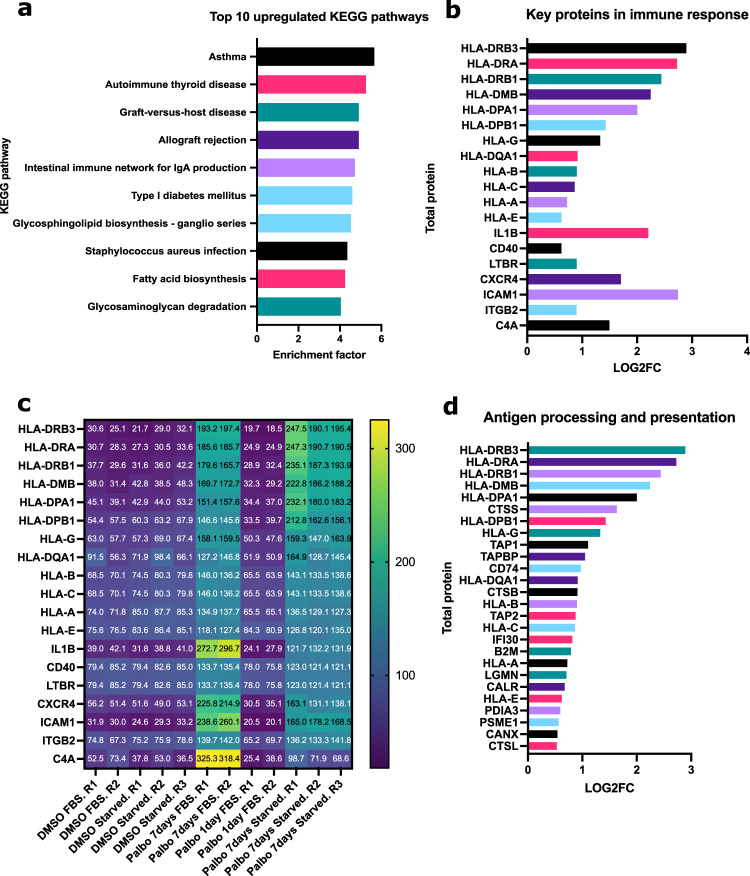
KEGG pathway enrichment of significantly upregulated proteins in 7 days treated Palbociclib MDA-MB-231 samples (FBS and starved samples) versus their respective DMSO control samples. (**a**) Top 10 most upregulated KEGG pathways as determined by a Fisher’s exact test (Benjamini-Hochberg FDR threshold = 0.05) on 1 sample t-test results where a column indicating upregulation (Log2FC > 0.5) or downregulation (Log2FC < −0.5) was added. (**b**) Log2FC of upregulated proteins in the top 10 upregulated KEGG pathways which are disease or immune-related (Asthma, Autoimmune thyroid disease, Graft-versus-host disease, Allograft rejection, Intestinal immune network for IgA production, Type I diabetes mellitus and Staphylococcus aureus infection). Log2FC of 7 days Palbociclib-treated samples (FBS and starved) compared to their respective DMSO controls (FBS and starved) was calculated using a one sample t-test and subsequent results were filtered for q-value < 0.05 using Perseus software^39^. **(c)** Total protein scaled abundances for the proteins in Top 10 upregulated KEGG pathways which are disease or immune-related. **(d)** Log2FC of proteins in the KEGG pathway named Antigen processing and presentation. Log2FC = Log2 Fold Change.

**Fig. 9:**
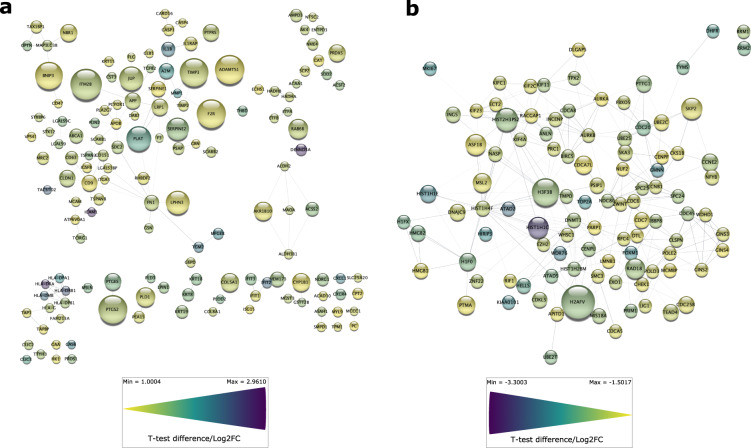
Physical network analysis of significantly upregulated and downregulated total protein upon Palbociclib treatment of MDA-MB-231 cells. **(a)** A physical network of upregulated proteins with a Log2 Fold Change (Log2FC) greater than 1 and Q-value < 0.01.**(b)** A physical network of downregulated proteins with a Log2FC less than −1.5 and Q-value < 0.01. One sample t-testing was performed using the Perseus software. Network mapping was performed using the Cytoscape application version 3.9.0^41^. All nodes were coloured with continuous mapping to represent Log2FC and were sized to represent Q-values.

To better understand the senescent-like state induced by Palbociclib in MDA-MB-231 cells, we used mass spectrometry to quantify the total proteome, phosphoproteome, and secretome of Palbociclib treated cells at day 1 and day 7 (Fig. 1). Palbociclib treatment resulted in striking morphological changes, such as the formation of flat and large cells (Fig. 2). We confirmed a high TMT labelling efficiency and efficient trypsin cleavage for all datasets as well as sample correlation (Figs. 3 and 4). Palbociclib treatment resulted in changes in the proteome and phosphoproteome which demonstrate failure to progress through G1/S via significant protein expression changes to cyclins and significant Rb dephosphorylation in response to CDK4/6 inhibition^4,5^ (Fig. 5). In addition, the cell cycle pathway is found amongst the most downregulated KEGG pathways which confirms cell cycle inibition in response to Palbociclib (Supplementary Table 1). Additionally, Palbociclib-treated MDA-MB-231 have well-recognised signatures of senescence including expression of GLB1 (*β*-galactosidase) and, loss of proliferation marker Ki67 and perinuclear LaminB1^23,27,28^ (Fig. 6). We validate the changed levels of these markers with immunofluorescence staining in addition to total protein abundances (Figs. 2 and 6). Statistical analysis depicts some interesting targets which are upregulated in response to Palbociclib, including adhesion molecules and components of the immune system (Fig. 7). Activation of the immune response is also reflected in the enrichment of KEGG pathways where proteins key to the immune response, e.g. MHC proteins, are amongst the drivers of the Top 10 upregulated pathways^34^ (Fig. 8). Furthermore, we note that significantly decreased expression of both RRM1 and RRM2, for example, could also indicate changes in the metabolic state of Palbociclib-treated cells^36,37^ (Fig. 7). Network visualization of upregulated proteins indicates the response of a number of key markers previously associated with the senescence phenotype in response to Palbociclib, such as the upregulation of GLB1 (*β*-galactosidase)^27^. In-depth analysis could allow the delineation of the metabolic profile of the Palbocicib-induced cell state in TNBCs, for example, as a number or central carbon metabolic pathways are shown to be upregulated. Figure 9 also depicts changes to histone molecules expression indicates that there may be potential epigenetic changes with downstream effects^38^. These datasets set the stage for the development of biomarkers to better track the effects of Palbociclib use in patients or stratify patients who will best benefit from Palbociclib therapy. Moreover, these datasets open therapeutic avenues for the development of strategies to enhance Palbociclib use.

## Methods

### Cell Cultures

#### Cell line maintenance

MDA-MB-231 were cultured in growth medium composed of Dulbecco’s Modified Eagle Medium (DMEM 1X) (4.5g/L D-Glucose, L-Glutamine and Pyruvate (Gibco), 10% heat-inactivated Fetal Bovine Serum (FBS) (Gibco) and 1% Penicillin/Streptomycin (Invitrogen). Cell cultures were maintained in 5% CO_2_, humidified incubators at 37 °C.

#### Passaging

For each passage, the existing growth medium was aspirated when cells were at 80% confluency. PBS (5ml) was added to wash the flask and was then aspirated.Subsequently, 2ml 0.25% Trypsin-EDTA (Gibco) was added and the flask was returned to the incubator for 5–10 mins to allow cell detachment. Once cells were detached, 5ml growth medium was added to the flask and cells were gently resuspended by pipetting. The entire volume of cells was added to a 15ml Falcon which was centrifuged at 1000rpm for 5 mins. Media was then aspirated whilst leaving the pellet intact. We resuspended the pellet in 2–5ml fresh growth medium. Trypan Blue (10 μL) was mixed with 10 μL cell solution and A 10 μL aliquot of the mixture was added to a Cell Countess glass slide (EVE,NanoEnTek). Cells in solution were counted using the Cell Countess (Invitrogen) and the required parameters were input on the Cell Countess (volume and concentration of cells required). The cell solution was resuspended in the required volume to achieve the cell concentration desired which was transferred to a new vessel.

### Proteomics/Phosphoproteomics drug treatment regimes (Samples 1–6)

For all experiments cells were seeded on T75 flasks at a concentration of 2 × 10^4^cells/ml in 14ml of growth medium per flask. Cells were allowed to settle and subsequently treated. Palbociclib-treated flasks (prefixed “Palbo” in all datasets) were treated with PD0332991 (Sigma, Lot #072M4735V), commonly known as “Palbociclib”, at a final concentration of 1 uM (1.4 μL/flask). DMSO control flasks were treated with an equivalent volume of DMSO (Sigma). Cell pellets were collected on 7 days and 1 day post-treatment for both Palbociclib-treated cells and DMSO controls (Table 1).

**Table 1:** Experimental setup overview.

Sample #	Name	Analysis	Treatment	FBS starved
1	DMSO control 7 days	Proteomics/Phosphoproteomics	DMSO	N
2	DMSO control 7 days	Proteomics/Phosphoproteomics	DMSO	N
3	Palbo 7 days	Proteomics/Phosphoproteomics	PD0332991 1 μM	N
4	Palbo 7 days	Proteomics/Phosphoproteomics	PD0332991 1 μM	N
5	Palbo 1 day	Proteomics/Phosphoproteomics	PD0332991 1 μM	N
6	Palbo 1 day	Proteomics/Phosphoproteomics	PD0332991 1 μM	N
7	Palbo 7 days	Secretome	PD0332991 1 μM	Y
8	Palbo 7 days	Secretome	PD0332991 1 μM	Y
9	Palbo 7 days	Secretome	PD0332991 1 μM	Y
10	DMSO control 7 days	Secretome	DMSO	Y
11	DMSO control 7 days	Secretome	DMSO	Y
12	DMSO control 7 days	Secretome	DMSO	Y

All samples collected and the analysis they were subjected to as indicated by the “Name” and “Treatment” columns. The “FBS starved” column indicates the replacement of standard growth medium containing foetal bovine serum (FBS) with FBS-free serum which is a standard procedure in secretome analysis. Y = yes. N= no.

### Secretome drug treatment regimes (Samples 7–12)

Cells were seeded on T75 flasks at 2 × 10^4^ cells/ml in 14 ml growth medium per flask. Cell cultures were allowed to settle and were then treated with PD0332991 (Sigma, Lot #072M4735V) at a final concentration of 1 μM. DMSO control flasks were once again treated with an equivalent volume of DMSO. In the 24 hours prior to sample collection, the flasks were washed with warm PBS to remove any serum. Growth medium was then replaced with an equivalent volume of FBS–free medium with 1 μM Palbociclib for treatment flasks and DMSO for control flasks (Table 1).

### Cell and growth media collection

For the samples collected for total protein analysis (samples 1-6), cell cultures were trypsinized and centrifuged into a cell pellet as when passaging. Cells were resuspended in 1ml cold PBS. The Cell Countess was used as previously, to count 1 × 10^6^ cells which were aliquoted in 1.5 ml Eppendorf tubes. These were centrifuged at 4 °C, 14,000 rpm for 5 mins to produce a pellet. Subsequently, PBS was aspirated and the samples were snap frozen on dry ice by spraying with ethanol. The samples were frozen at –80 °C.

For secretome analysis (samples 7–12), growth medium was filtered through 0.2 μm filters and collected in 50 ml Falcon tubes. FBS-free growth medium (10ml) was then used to wash each T75 flask and added to the same 50 ml Falcon tube. The collected media was frozen at −80 °C.

### Sample preparation for proteomics analysis of cell lines and secretome

Cell pellets were lysed in 150 μL lysis buffer of 1% Sodium Deoxycholate (SDC), 100 mM Triethylammonium Bicarbonate (TEAB), 10% Isopropanol, 50 mM NaCl and Halt protease and phosphatase inhibitor cocktail (100X) (Thermo, #78442) on ice, assisted with pulsed probe sonication for 15 sec and followed by heating at 90 °C for 5 min and re-sonicated for 5 sec. Protein concentration was measured with the Quick Start™ Bradford Protein Assay (Bio-Rad) according to manufacturer’s instructions. Protein aliquots of 60 μg were reduced with 5 mM Tris-2-Carboxyethyl Phosphine (TCEP) for 1 h at 60 °C and alkylated with 10 mM Iodoacetamide (IAA) for 30 min in dark. Proteins were digested overnight with trypsin at final concentration 75 ng/μL (Pierce) and peptides were labelled with the TMTpro reagents (Thermo) according to manufacturer’s instructions. The pooled sample was acidified with 1% formic acid to remove precipitated SDC by centrifugation and supernatant was SpeedVac dried. For the secretome analysis, the collected culture media were concentrated with the Amicon Ultra 15mL 3K MWCO ultrafiltration devices, the proteins were precipitated with trichloroacetic acid (TCA) and washed with cold acetone. Protein pellets were solubilized in lysis buffer and processed as above but using the TMT10plex reagents.

### High-pH Reversed-Phase peptide fractionation and phosphopeptide enrichment

For the analysis of cell pellets and secretome, the TMT labelled peptides were fractionated with high-pH Reversed-Phase (RP) chromatography using the XBridge C18 column (2.1 x 150 mm, 3.5 μm, Waters) on a Dionex UltiMate 3000 HPLC system. Mobile phase A was 0.1% v/v ammonium hydroxide and mobile phase B was acetonitrile, 0.1% v/v ammonium hydroxide. The peptides were fractionated at 0.2 mL/min with the following gradient: 5 mins at 5% B, up to 12% B in 3 min, for 32 min gradient to 35% B, gradient to 80% B in 5 min, isocratic for 5 mins and re-equilibration to 5% B. Fractions were collected every 42 sec, combined in 30 (cell pellets) and 12 (secretome) fractions and SpeedVac dried.

Phosphopeptide enrichment was performed for 24 fractions (cell pellets) with the High-Select™ Fe-NTA Phosphopeptide Enrichment Kit (Thermo) using a customized protocol in a well plate array format. A volume of 50 μL resin/buffer was transferred on top of 10 μL filter tips that were fitted on a 96-well plate using a tip rack adapter. The resin was washed three times with 40 μL wash/binding solution and centrifugation at 500 g for 1 min. Peptides were reconstituted in 30 μL wash/binding solution and were loaded onto the tip-columns with the resin. After 30 min, the flow-through from three washes with wash/binding solution were collected in a clean 96-well plate with centrifugation at 500 g for 1 min each time. Phosphopeptides were eluted twice with 40 μL elution buffer in a clean 96-well plate with centrifugation at 500 g for 1 min, transferred in glass vials (Waters, P/N 186005669CV) and SpeedVac dried. The flow-through solutions were dried and kept for total proteome analysis.

### LC-MS analysis

LC-MS analysis was performed on a Dionex UltiMate 3000 UHPLC system coupled with the Orbitrap Lumos Mass Spectrometer (Thermo Scientific). Peptides were loaded onto the Acclaim PepMap 100, 100 μm × 2 cm C18, 5 μm, trapping column at flow rate 10 uL/min. Samples were analysed with the EASY-Spray C18 capillary column (75 μm × 50 cm, 2 μm) at 50 °C. Mobile phase A was 0.1% formic acid and mobile phase B was 80% acetonitrile, 0.1% formic acid. The separation method was: for 90 min gradient 5%-38% B, for 10 min up to 95% B, for 5 min isocratic at 95% B, re-equilibration to 5% B in 5 min, for 10 min isocratic at 5% B at flow rate 300 nL/min. MS scans were acquired in the range of 375–1,500 m/z with mass resolution of 120 k, AGC 4×105 and max IT 50 ms. Precursors were selected with the top speed mode in 3 sec cycles and isolated for HCD fragmentation with quadrupole isolation width 0.7 Th. Collision energy was 36% (TMTpro) or 38% (TMT10plex) with AGC 1×105 and max IT 86 ms at 50 k resolution. Targeted precursors were dynamically excluded for further fragmentation for 45 seconds with 7 ppm mass tolerance. The phosphopeptide enriched samples were analysed as above with the following differences: 60 min gradient 5%-38% B, HCD MS2 with max IT 100 ms and 30 sec dynamic exclusion.

### Database search and quantification

The mass spectra were analysed in Proteome Discoverer 2.4 (Thermo Scientific) with the SequestHT search engine for peptide identification and quantification. The precursor and fragment ion mass tolerances were 20 ppm and 0.02 Da respectively. Spectra were searched for fully tryptic peptides with maximum 2 miss-cleavages. TMTpro (cell pellets) or TMT6plex (secretome) at N-terminus/K and Carbamidomethyl at C were selected as static modifications. Oxidation of M, Deamidation of N/Q as well as Phosphorylation of S/T/Y for the IMAC samples only were selected as dynamic modifications. Spectra were searched against reviewed UniProt human protein entries, peptide confidence was estimated with the Percolator node and peptides were filtered at q-value < 0.01 based on decoy database search. The reporter ion quantifier node included a TMT quantification method with an integration window tolerance of 15 ppm. Only peptides with average reporter signal-to-noise > 3 were used and phosphorylation localization probabilities were estimated with the IMP-ptmRS node.

### Immunofluorescence staining

Cells were plated in 384-well plate at a concentration of 400 cells/well. Cells were allowed to attach and subsequently treated with 1uM PD0332991 (“Palbociclib”, Sigma, Lot #072M4735V). After 7 days of treatment, cells were fixed with 4% PFA (Thermo) and incubated for 12 mins. Wells were washed twice with PBS (supplemented with NaN_3_). Subsequently, 20 μL 0.2% Triton-X solution was added per well and allowed to incubate for 12 mins. Wells were washed twice with PBS (supplemented with NaN_3_) and 20 μL 2% BSA was added and incubated for 1 hr. All steps were performed at ambient temperature. Wells were aspirated. Antibody solution mix was made up with 0.5% BSA, 0.02% Triton-X and PBS (with NaN_3_). In the relevant volume of antibody solution, primary antibodies were added at the required concentration (Table 2). Plates were enclosed in Parafilm and incubated at 4 °C overnight. Wells were subsequently washed thrice with PBS (with NaN_3_). Secondary antibodies were added in a concentration of 1:1000 in antibody solution mix and incubated for 2hrs at ambient temperature. Wells were washed thrice with PBS (with NaN_3_) and Hoescht dye was added at a concentration of 1:1000 in antibody solution mix. Plates were incubated for 10 mins at ambient temperature and then washed once with PBS (with NaN_3_). Wells were left in PBS (with NaN_3_) and stored at 4 °C. Subsequently, imaging was performed using the Opera high-throughput microscope (PerkinElmer).

**Table 2:** Primary, secondary antibodies and dyes for immunofluorescence staining.

Antibodies/Dyes	Species	Dilution	Manufacturer
**pRb(807/811)**	Rabbit	1:1000	Cell signaling (Cat 8516S)
**KI67**	Rabbit	1:50	Abcam (#16667)
**LMNB1**	Rabbit	1:1000	Abcam (#16048)
**Tubulin Alpha**	Rat	1:1000	BioRad (Ref MCA78G)
**Alexa Fluor 488 Phalloidin**	—	1:1000	Invitrogen (Cat A-12379)
**Alexa Fluor 568 Phalloidin**	—	1:1000	Invitrogen (Cat A-12380)
**Goat anti-Rat IgG (H+L) Cross-Adsorbed Secondary Antibody, Alexa Fluor 568**	Rat	1:1000	Invitrogen (Cat A-11077)
**F(ab’)2-Goat anti-Rabbit IgG (H+L) Cross-Adsorbed Alexa Fluor 647**	Rabbit	1:1000	Invitrogen (Cat A-21246, Lot 2147629)
**Goat anti-Rabbit IgG (H+L) Secondary antibody Highly Cross-Adsorbed, Alexa Fluor 488**	Rabbit	1:1000	Invitrogen (Cat A-11034)
**Goat anti-rat IgG (H+L) Secondary antibody Cross-Adsorbed, Alexa Fluor 647**	Rat	1:1000	Invitrogen (Cat A-21247)
**Hoescht 33258**	—	1:1000	Invitrogen (Cat H3569)

### Preliminary analysis

Statistics was performed using the Perseus Software^39^. VolcanoseR (mirror R2) was used for visualization of one sample t-test and extraction of top 50 hits in all datasets^40^.The Cytoscape application version 3.9.0 was used for network analysis^41^. In addition, the Phantasus server was used for creating a similarity matrix (Pearson correlation) and hierarchical clustering (Euclidean distance with complete linkage) with the total protein scaled abundances for technical validation (https://artyomovlab.wustl.edu/phantasus/). Principal component analysis (PCA) was performed using the python package bioinfokit with scaled abundance values of the total proteome.

## Data Records

Raw mass spectrometry data files and post-processed, scaled abundance Excel sheets for total proteome, phosphoproteme and secretome have been deposited to the ProteomeXchange Consortium via the PRIDE partner repository^42,43^ under the dataset identifier PXD030407^44^.

## Technical Validation

### Reproducibility of Replicates

All samples were subjected to PCA. The first 3 principal components (PCs) were plotted to show clustering of samples under the same conditions (Fig. 3a). A biplot was also used to illustrate the close association between the “Palbo 7 days FBS” and “Palbo 7 days starved” samples, as well as the degree of difference from their respective DMSO control samples (Fig. 3b). Replicates for all conditions were subjected to a similarity matrix (Pearson correlation). Subsequently, hierarchical clustering using a Euclidean metric (complete linkage) was applied on the similarity matrix which showed that all replicate samples cluster together on the matrix (Fig. 3c).

### Mass spectrometry

TMT labeling efficiency was estimated by Mascot search of 10,000 representative MS2 scans with TMT selected as variable modification (at peptide n-term and K). The estimated labelling efficiencies were >99% for the total proteome/phosphoproteome and 98% for the secretome data. Figure 4 shows the percentages of trypsin missed cleavages (10%) indicating efficient digestion in both sample types.

## Usage Notes

Mass Spectrometry (MS) datafiles are available to users to allow different proteome and phospho-proteome analyses in order to delineate the drug response to Palbociclib in a model TNBC cell line. Figure 9 shows the upregulation of a number of key senescence markers which can act as controls for our dataset. The network nodes are coloured by Log2 Fold Change (Log2FC) and sized by q-value to indicate significance. This type of analysis can reveal a number of pathways which can be used as controls for response to Palbociclib, e.g. cell cycle downregulation, but can also reveal changes which may suggest the induction of senescence. For example, FOXM1, which is thought to be an inhibitor of senescence, is shown to be downregulated^45^. Figure 9 also depicts changes to histone molecules expression indicates that there may be potential epigenetic changes with downstream effects^38^. In addition, a number of subnetworks could reveal further changes due to Palbociclib, such as an IFN response, metalloproteinases (e.g.MMP1) and immune system activation. These observations are also corroborated by annotation of the datasets using resources such as the KEGG pathways database^46^ as well as Gene Ontology terms^47,48^ which could also provide further evidence of a wider-acting network of changes including immune activation, metabolic and transcriptional changes (Fig. 8 and Supplementary Table 1). The metabolic reprogramming associated with malignancy induction has been suggested to be either reversed or enhanced in a senescent cell sub-population with evidence towards both being found in the literature^49,50^. Preliminary, statistical analysis shows upregulation and downregulation of proteins and phosphorylation states which could indicate the induction of a range of programmes which lead to the morphological and phenotypic changes associated with Palbociclib treatment in TNBCs (Fig. 7). Notably, TACSTD2 upregulation, which is also known as Trop-2, could point to a potential combination therapy as anti-Trop-2 antibodies and antibody-drug conjugates have shown success in TNBCs^51–54^. Lastly, feature extraction algorithms could be used to enquire further into the associations between morphological changes and senescence reprogramming.

## Supplementary information

Supplementary Table 1

## Data Availability

Statistics (one sample t-test) was performed using the Perseus software version 1.6.14.0^39^. PCA was performed and plotted using the python bioinfokit package^55^. Pearson correlation, hierarchical clustering analysis and subsequent similarity matrix were performed and created using the Phantasus software version 1.11.0 (https://artyomovlab.wustl.edu/phantasus/). Volcano plots were created using VolcaNoseR (mirror R2)^40^. Network analysis was performed using the String application on Cytoscape version 3.9.0^41^. Heatmap and bar charts were created using GraphPad Prism version 9.2.0. Images were stored on Perkin Elmer Columbus server version 2.9.1. No custom code was used to process the data in this manuscript.
